# BRE facilitates skeletal muscle regeneration by promoting satellite cell motility and differentiation

**DOI:** 10.1242/bio.012450

**Published:** 2016-01-06

**Authors:** Lihai Xiao, Kenneth Ka Ho Lee

**Affiliations:** Stem Cell and Regeneration Thematic Research Programme, School of Biomedical Sciences, Chinese University of Hong Kong, Hong Kong

**Keywords:** BRE, Knockout mice, Muscle satellite cells, Skeletal muscle regeneration, Cell migration

## Abstract

The function of the *Bre* gene in satellite cells was investigated during skeletal muscle regeneration. The tibialis anterior leg muscle was experimentally injured in *Bre* knockout mutant (BRE-KO) mice. It was established that the accompanying muscle regeneration was impaired as compared with their normal wild-type counterparts (BRE-WT). There were significantly fewer pax7^+^ satellite cells and smaller newly formed myofibers present in the injury sites of BRE-KO mice. *Bre* was required for satellite cell fusion and myofiber formation. The cell fusion index and average length of newly-formed BRE-KO myofibers were found to be significantly reduced as compared with BRE-WT myofibers. It is well established that satellite cells are highly invasive which confers on them the homing ability to reach the muscle injury sites. Hence, we tracked the migratory behavior of these cells using time-lapse microscopy. Image analysis revealed no difference in directionality of movement between BRE-KO and BRE-WT satellite cells but there was a significant decrease in the velocity of BRE-KO cell movement. Moreover, chemotactic migration assays indicated that BRE-KO satellite cells were significantly less responsive to chemoattractant SDF-1α than BRE-WT satellite cells. We also established that BRE normally protects CXCR4 from SDF-1α-induced degradation. In sum, BRE facilitates skeletal muscle regeneration by enhancing satellite cell motility, homing and fusion.

## Abstract (precis)

**Summary:** BRE facilitates skeletal muscle regeneration by promoting satellite cell motility and differentiation, probably by protecting CXCR4 from degradation.

## INTRODUCTION

Skeletal muscle accounts for approximately 40-50% of the human body's mass and it's the most abundant tissue. The main function of skeletal muscle is to maintain posture and generate movement of the body. Besides bone marrow, skeletal muscle exhibits a tremendous regenerative capacity in response to injuries (Collins et al., 2005). The main regenerative potential of skeletal muscle is derived from a small population of stem cells found outside muscle fibers called satellite cells. Under normal condition, satellite cells are maintained in a quiescent state and accounted for only about 2-7% of the total nuclei observed in adult skeletal muscles (Abou-Khalil et al., 2010). However, this percentage will increase to about 10-15% in response to muscle injury, when quiescent satellite cells are induced to proliferate. The satellite cells will migrate to the injury site, fuse with each other and differentiate to create new myofibers. The newly-formed myofibers will further fuse with existing intact myofibers to repair the damaged muscle. The extent of this bioactivity is depending on the severity of injury. *In vitro*, it is possible to follow the chronological and dynamic process of satellite cell migration, differentiation and fusion (O'Connor et al., 2007; Bae et al., 2008). Many proteins have been now identified to be important in the regulation of satellite cell migration which includes: fibroblast growth factor (FGF), hepatocyte growth factor (HGF) and IL-4 (Lluri and Jaworski, 2005; Lafreniere et al., 2006). Components of the extracellular matrix (such as fibronectin and laminin) and chemokines (such as SDF-1α) also play important roles in regulating these biological processes (Griffin et al., 2010).

The *Bre* (brain and reproductive organ-expressed protein) gene was originally identified as a gene that was responsive to cellular DNA damage and retinoic acid treatment (Li et al., 1995). Normally, this gene was found extensively expressed in brain, testicular and ovarian tissues, hence it was named *Bre*. Subsequent studies have revealed that *Bre* was expressed in most of the organs including the skeletal muscles. The *Bre* gene encodes a 1.9 kb full length mRNA and transcribes a highly conserved 383 amino acid protein, with the molecule weight of 44 kDa. The protein contains no known functional domains. BRE protein, also known as TNFRSF1A modulator or BRCC45, is normally expressed in the cytoplasm but under stress and pathological conditions, it is also found in the nucleus. In the nucleus, BRE is a component of the BRCA1-RAP80 complex and acts as an adaptor protein linking NBA1 with the rest of the protein complex. Following DNA damage, the complex exhibited E3 ligase activity so as to enhance cell survival (Dong et al., 2003). In the cytoplasm, BRE is also a component of the BRISC (BRCC36 Isopeptidase complex) complex. During apoptotic induction, BRE will bind to the cytoplasmic region of TNF-R1 (Gu et al., 1998), Fas (Li et al., 2004) and DISC (Patterson et al., 2010) to protect cells from apoptosis and enhance cell survival.

In this study, we examined the function of BRE in skeletal muscles since nothing is known about its normal function *in vivo*. We generated BRE knockout (BRE-KO) mice and studied how the absence of BRE affected skeletal muscle regeneration. We also isolated satellite stem cells from wild-type (BRE-WT) and BRE-KO mouse skeletal muscles and compared the ability of these cells to migrate, fuse and differentiate. Using the skeletal muscle model, we maximize our understanding of BRE in these fundamental processes. In addition, the satellite stem cells also present the opportunity to investigate the developmental potency of satellite stem cells in the absence of BRE expression.

## RESULTS

### *Bre* expression in BRE-WT and BRE-KO muscles

**Fig. 1.**
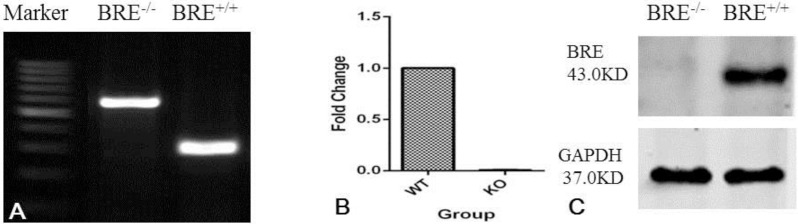
**Validation of *Bre* null mutation in the skeletal muscles of BRE-KO mice.** (A) PCR genotyping showing exon 3 of the *Bre* gene has been deleted in *Bre^−/−^* tissue. RT-qPCR (B) and Western blot (C) analysis also confirm the *Bre^−/−^* skeletal muscles do not express *Bre* mRNA and protein, respectively. GAPDH was used as internal control.

BRE-KO mice were generated by crossing male TNAP^Cre/+^ mice with female BRE^fx/fx^ mice according to the breeding strategy illustrated in Fig. S1. We first validate that the *Bre* gene was completely knocked out at the DNA, mRNA and protein levels in our BRE-KO mice. Skeletal muscle cells were harvested from BRE-WT and BRE-KO mice and used for PCR, Real-time RT-PCR and western blot analysis. The PCR genotyping show exon 3 has been deleted from the full length *Bre* gene (Fig. 1A). The RT-qPCR results revealed that BRE-WT skeletal muscle cells could express *Bre* mRNA but not by BRE-KO cells (Fig. 1B). Similarly, western blot show BRE-WT skeletal muscle cells could express BRE protein not BRE-KO cells (Fig. 1C). We found that newborn BRE-KO mice were grossly indistinguishable from BRE-WT mice. We x-rayed the older mice and again found no difference between the skeleton of BRE-KO and BRE-WT mice (Fig. S2).

### Skeletal muscle regeneration is delayed in BRE-KO mice

**Fig. 2.**
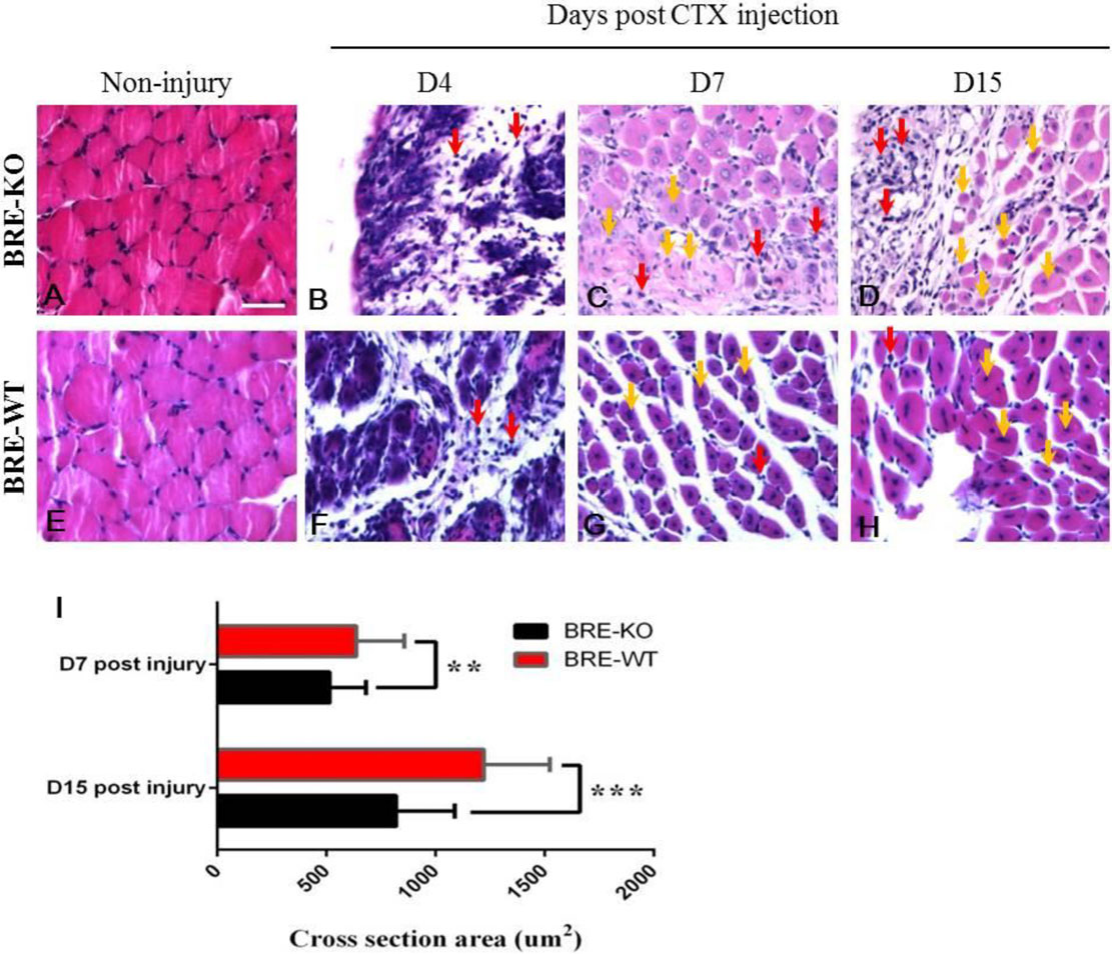
**Tibialis anterior muscles of BRE-KO (A-D) and BRE-WT (E-H) mice chemically damaged with cardiotoxin and allowed to regenerate for 4-15 days.** Histological sections stained with H&E showed there were necrotic myofibers and numerous small mononucleated lymphocytes (red arrows) present at the injury sites in both BRE-KO mice (B) and BRE-WT (F). At day 7 post-injury, almost of the damaged myofibers have disappeared and were replaced by small newly-formed myofibers (yellow arrows) with centrally localized nuclei in BRE-WT muscles (G). In BRE-KO muscles, there were both newly formed myofibers and myofibers in the process of being formed (yellow arrows). The latter was less evident than in corresponding BRE-WT injured muscles. There were still numerous degenerating myofibers present in the BRE-KO injury site (C). At day 15 post-injury, there were more newly regenerated myofibers present in both BRE-WT (H) and BRE-KO (D) muscles. (I) Bar chart showing the average cross section area of the newly-formed myofibers in 7 and 15 day post-injury muscles. Only myofibers with centrally localized nuclei were measured and 3 mice for each genotype were analyzed. The results showed newly-formed myofibers were significantly smaller in BRE-KO muscles than in BRE-WT muscles, day 7 (**P<0.01) and day 15 (***P<0.001) post-injury. Data are presented as mean±s.e.m. Scale bar=50 μm.

We investigated whether the *Bre* gene influences skeletal muscle regeneration. The tibialis anterior muscle of both BRE-WT and BRE-KO mice were injected with CTX and then harvested for analysis 4, 7 and 15 days post-injection. Between day 1-4 post-injury, there were necrotic myofibers and numerous small mononucleated lymphocytes present at the injury sites of both BRE-WT (Fig. 2F) and BRE-KO mice (Fig. 2B). In BRE-WT muscles, almost all of the damaged myofibers have disappeared and were replaced by small newly-formed myofibers, at day 7 post-injury (Fig. 2G). The nuclei in these newly-regenerated myofibers are centrally localized within the fibers. In the BRE-KO injury muscle site, there were still numerous degenerating myofibers but also small newly-formed myofibers and myofibers in the process of being formed (Fig. 2C). At day 15 post-injury, there were proportionally more newly regenerated myofibers increased in both BRE-WT (Fig. 2H) and BRE-KO (Fig. 2D) injured muscles. However, there were still unrepaired muscle areas visible in the BRE-KO group. We calculated that the average cross section area of the newly-formed myofibers in day 7 and day 15 post-CTX treated muscles. It was determined that, at these two time points, the newly-formed myofibers in BRE-KO muscles were significantly smaller in BRE-WT muscles (Fig. 2I).

**Fig. 3.**
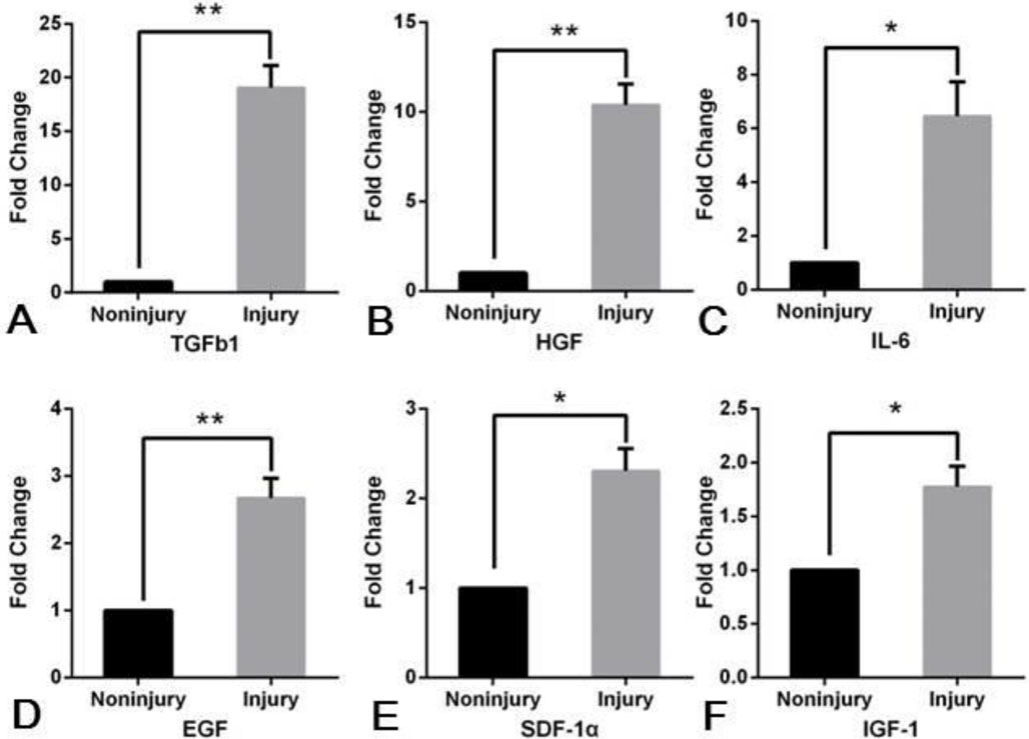
**RT-qPCR analysis of the muscle injury site in BRE-WT mice, 4 days post-injury.** The results show a significantly increase in TGFβ1 (A), HGF (B), IL-6 (C), EGF (D), SDF-1α (E) and IGF-1 (F) expression compared with the uninjured contralateral control muscles. Data are presented as mean±s.e.m. Significant difference, **P<0.01 and *P<0.05. *N*=3.

It has been reported that, after muscle injury, the inflammatory response is activated and this lead to the destruction of the damaged muscle fibers. Neutrophils, leukocytes and macrophages migrated to the injury site, to initiate myolysis and remove cellular debris. During this process, growth factors and cytokines are secreted which regulates the activation, proliferation, differentiation and migration of satellite stem cells. In this context, we investigated the expression of key cytokines before and after muscle injury. We established that normal injured muscles expressed significantly higher levels of TGFβ1, HGF, IL-6, EGF, SDF-1α and IGF-1, 4 days post-injury (Fig. 3).

**Fig. 4.**
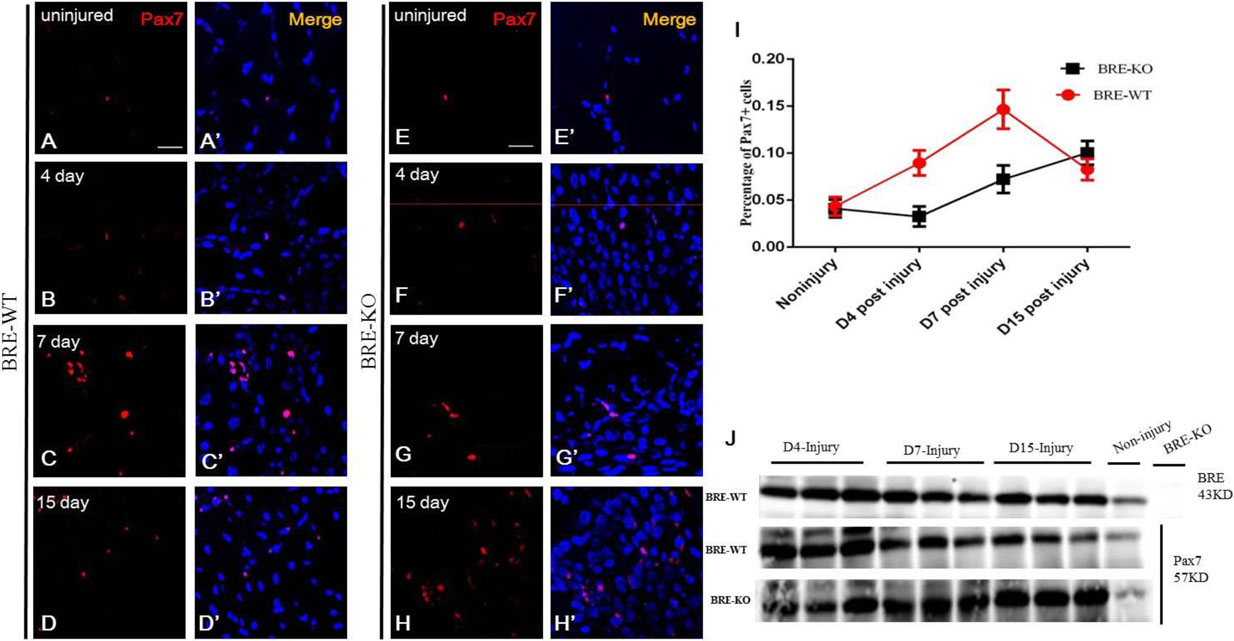
**Immunofluorescent staining showing the presence of pax7^+^ cells in BRE-WT (A-D) and BRE-KO (E-H) tibialis anterior before and after cardiotoxin-induced injury.** (I) Chart showing there were 9% pax7^+^ satellite cells present in BRE-WT injury site while only 3.3% for BRE-KO after 4 days post-injury. At day 7, this percentage increased to 14.7% and 7.2% in the BRE-WT and BRE-KO muscle injury sites, respectively. At day 15, in the BRE-WT muscle, the percentage of pax7^+^ satellite cells significantly drops to about 8.3%. By contrast, the percentage of pax7^+^ satellite cells present in BRE-KO injury site increased to 10% from 7%. J: Western blot analysis of BRE and pax7 expression before and after muscle injury. Both BRE and pax7 expression was up-regulated after muscle injury. Three mice for each genotypes were counted. Data are presented as mean±s.e.m. Scale bar=20 μm.

Next, we estimated and compared the percentage of pax7^+^ satellite cells present in the injury sites of BRE-WT and BRE-KO muscles (Fig. 4). Immuno-fluorescent staining revealed that 4 days after injury, there were 9% pax7^+^ satellite cells present in BRE-WT injury site and 3.3% in BRE-KO injury site. Seven days after injury, this percentage increased to 14.7% and 7.2% in BRE-WT and BRE-KO muscle injury site, respectively. At day 15 when muscle regeneration was almost completed in BRE-WT muscle, the percentage of pax7^+^ satellite cells significantly decreased to about 8.3%. By contrast, the percentage of pax7^+^ satellite cells present in BRE-KO injury site increased to 10% from 7%. The results indicate that the injured BRE-KO muscles do not regenerate as efficiently as BRE-WT muscles.

### *Bre* does not affect satellite cell morphology

**Fig. 5.**
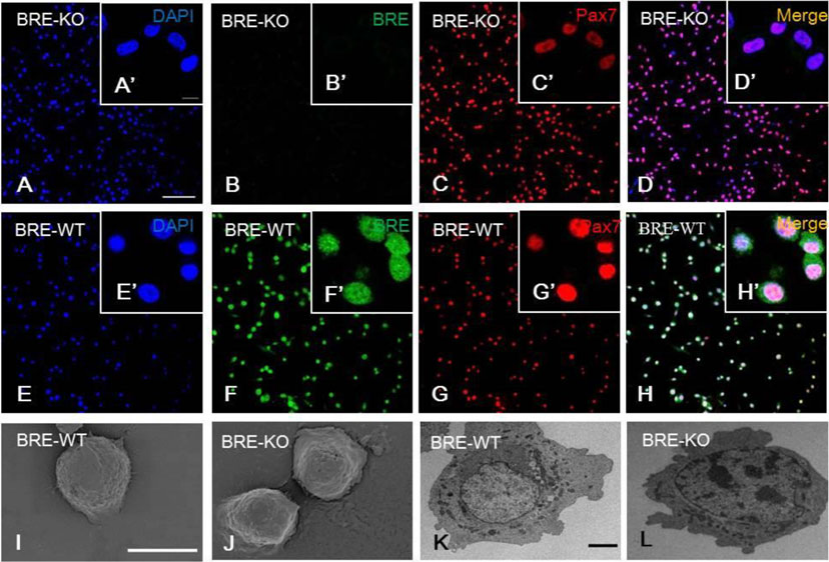
**BRE-KO (A-D′) and BRE-WT (E-H′) satellite cells immunofluorescently stained with BRE and pax7 antibodies.** The staining show BRE is expressed in the nucleus and cytoplasm of BRE-WT satellite cells (F). In the absence of BRE, the satellite cells are still capable of expressing pax7 (C). Under the scanning electron microscope, the morphology of both BRE-WT (I) and BRE-KO (J) satellite cells appeared rounded. Under the transmission electron microscope, the satellite cells show a high nuclear to cytoplasmic ratio in both BRE-WT (K) and BRE-KO (L) satellite cells. There was no morphological difference between the two groups. Scale bars, 100 μm for A-H, 10 μm for A′-H′, 100 μm for I-J and 20 μm for K-L.

We have isolated satellite cells from BRE-WT and BRE-KO EDL muscles using the single myofiber culture method and serial purification on uncoated culture dishes (Fig. S3). We immuno-fluorescently stained the purified satellite cell cultures with pax7 antibody and found about 95% of the cells were pax7^+^ in both BRE-KO (Fig. 5A-D) and BRE-WT (Fig. 5E-H) groups. These satellite cells were processed for SEM and TEM examinations. Under the scanning electron microscope, the morphology of BRE-WT and BRE-KO satellite cells appeared rounded (Fig. 5I and J, respectively). These cells have a high nuclear to cytoplasmic ratio both in BRE-WT (Fig. 5K) and BRE-KO (Fig. 5L) groups as revealed under the transmission microscope, but there was no obvious morphological difference between the two groups.

### BRE is required for satellite cell fusion

**Fig. 6.**
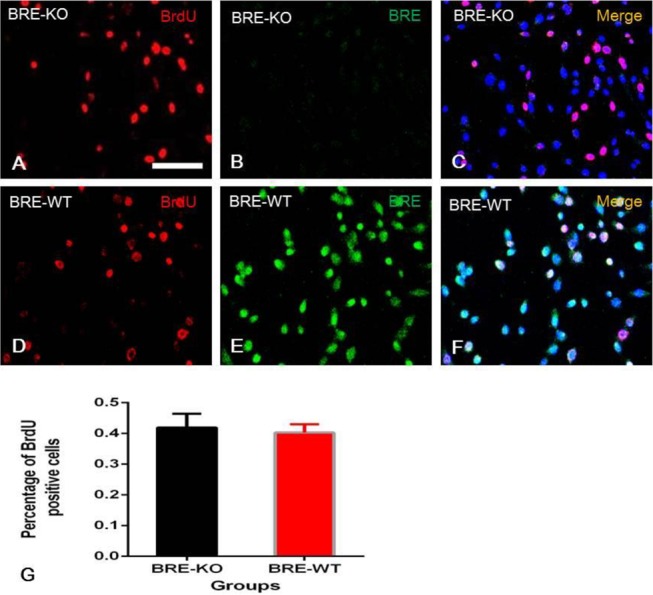
**Satellite cells were pulse labelled with BrdU and then double immune-fluorescently stained with BrdU and BRE antibodies.** BrdU^+^ satellite cells were present in both BRE-KO (A-C) and BRE-WT (D-F) cultures. (G) Bar chart showing there was no significant difference in the number of BrdU^+^ satellite cells in BRE-KO and BRE-WT cultures. Data were presented as mean±s.e.m. Scale bar=100 μm.

We investigated whether the absence of BRE expression affected satellite cell proliferation. To address this question, we pulse labelled BRE-KO and BRE-WT satellite cells with 10 μM BrdU for 3 h. The cultures were then double immunofluorescently stained with BrdU and BRE antibodies. Under the confocal microscope, we determined that BrdU^+^ satellite cells were present in both BRE-KO (Fig. 6A) and BRE-WT (Fig. 6D) cultures. We calculated and compared the percentage of proliferating cells present but found no significant difference between BRE-KO and BRE-WT satellite cell cultures (Fig. 6G). It appears that the absence of BRE do not affect satellite cell proliferation.

**Fig. 7.**
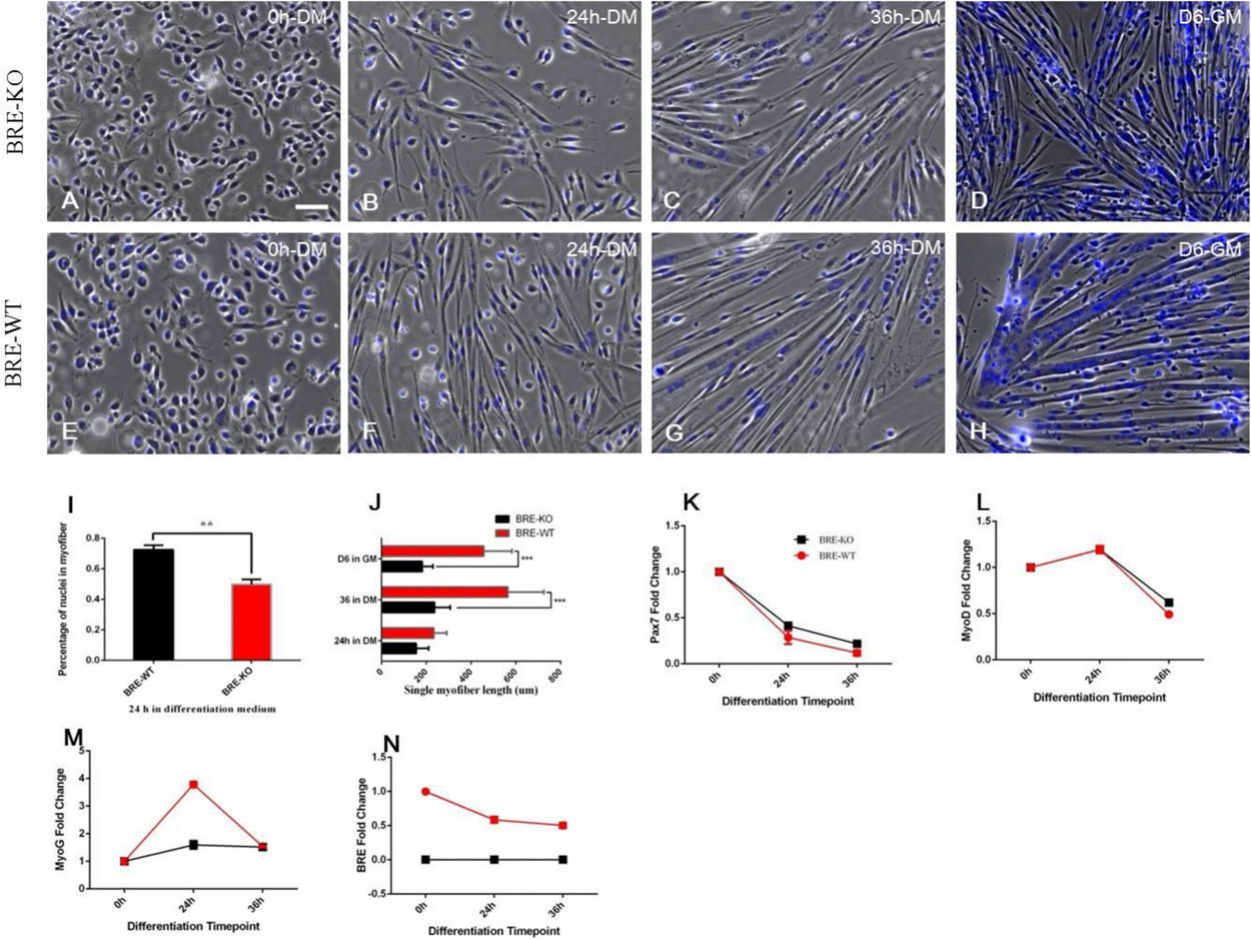
**BRE-KO (A-D) and BRE-WT (E-G) satellite cells cultured in the presence of myogenic differentiation medium (DM) at 0 h (A,E), 24 h (B,F) 36 h (C,G) and growth medium (GM) at 6 days (D,H).** In the presence of DM, the satellite cells were able to align with each other and fused to form myofibers in both BRE-KO and BRE-WT cultures (B-G). However, the BRE-KO satellite cell fusion index was determined to be 49.85±3.1% which was significantly lower than BRE-WT (72.47±3.0%, **P<0.01) (I). The average length of a single myofiber was measured after 36 h culture in DM (J). BRE-KO myofibers were found to be significantly shorter than BRE-WT myofibers (237.03±70 μm vs 564.05±161 μm, respectively, ***P<0.001). For satellite cells maintained in the presence of GM for 6 days, the average length of BRE-KO myofibers was again significantly shorter than BRE-WT myofibers (182.81±48 μm vs 456.11±127 μm, respectively, ***P<0.001). RT-qPCR analysis revealed that there were no differences in *pax7* (K) and *MyoD* (L) expression between BRE-KO and BRE-WT satellite cells cultured for 24-36 h in DM. However, BRE-WT satellite cells expressed significantly higher levels of *MyoG* (M) at 24 h than BRE-KO satellite cells. The data are presented as mean±s.e.m. Scale bar=100 μm.

Next, we examined whether the absence of BRE affected satellite cell fusion to form myofibers. We exposed BRE-KO and BRE-WT satellite cells to differentiation medium, composed of DMEM F12 supplemented with 10% horse serum and 1% PS, for 24-36 h. We found that satellite cells have aligned with each and fused to form myofibers after 24 h induction in myogenic differentiation medium, in both BRE-KO (Fig. 7B) and BRE-WT (Fig. 7F) cultures. However, the BRE-KO satellite cell fusion index (49.85±3.1%) was significantly lower than BRE-WT (72.47±3.0%) (Fig. 7I). Furthermore, the average length of BRE-KO myofibers is significantly shorter 237±70 μm (36 h treatment) and 183±48 μm (6 day treatment) than BRE-WT myofibers 564±161 μm (36 h treatment) and 456±127 μm (6 day treatment) (Fig. 7J). The results imply that BRE is necessary for complete fusion of satellite cells. Since BRE-KO satellite cell fusion was affected, RT-qPCR was performed to establish how expression of key myogenic differentiation genes was correspondingly affected. The results revealed that there were no differences in *pax7* (Fig. 7K) and *MyoD* (Fig. 7L) expression between BRE-KO and BRE-WT satellite cells, 24-36 h after exposure to differentiation medium. However, we found that BRE-WT satellite cells expressed higher levels of *MyoG* (Fig. 7M) at 24 h than corresponding BRE-KO satellite cells.

### Osteogenic differentiation potential is repressed in BRE-KO satellite cells

**Fig. 8.**
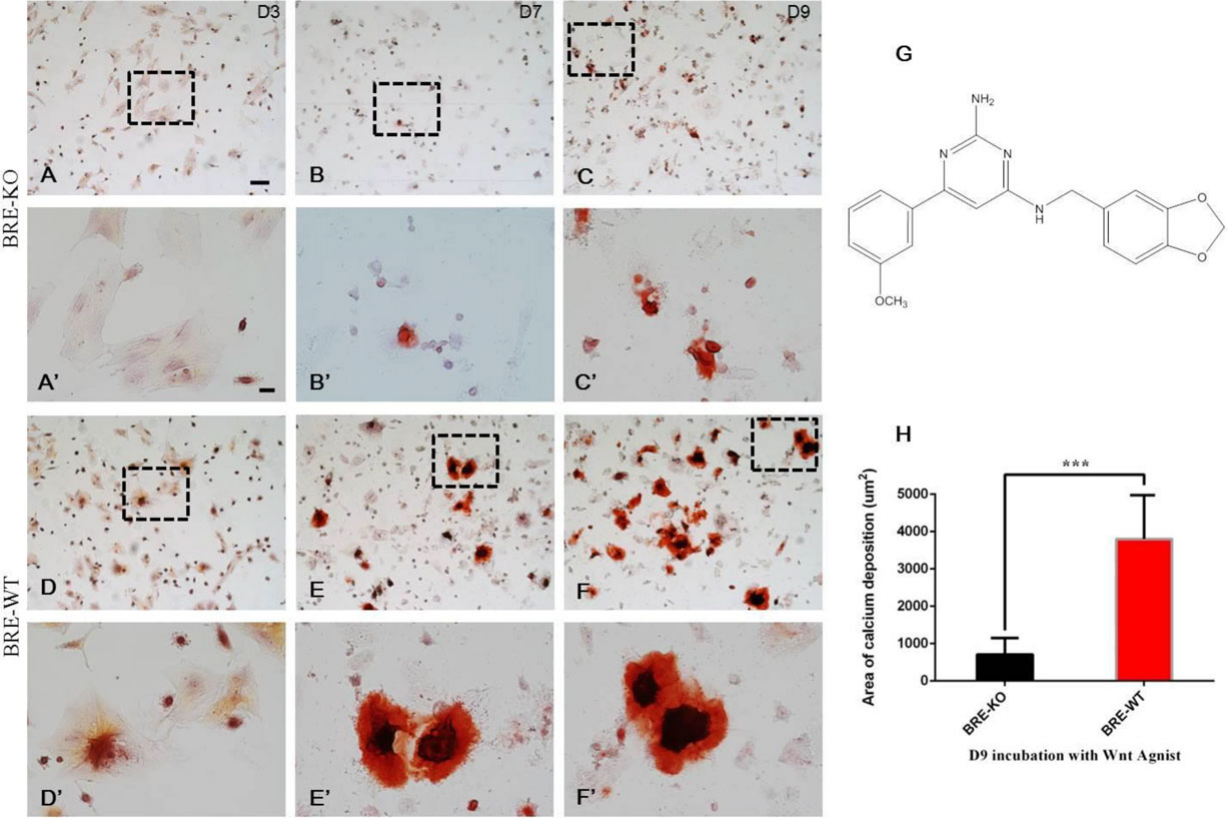
**BRE-KO (A-C) and BRE-WT (D-F) satellite cells were treated with a Wnt agonist (G, a small osteogenic-inducing molecule) for 3-9 days, to test these cells' osteogenic potentials.** (A′-F′) are higher magnification of the framed regions in images of (A-F). Alizarin Red staining revealed that BRE-WT satellite cells began to undergo osteogenic differentiation after 3 days treatment (D′). BRE-WT satellite cells produced significantly more calcium depositions at day 7 (E′) and 9 (F′) compared with BRE-KO satellite cells (B′ and C′, respectively). (H) Statistical analysis revealed that the average area occupied by osteoblasts and calcium deposits in BRE-WT cultures was significantly larger than in BRE-KO cultures at day 9 (***P<0.001). Data are presented as mean±s.e.m. Scale bars, 100 μm for A-F, 20 μm for A′-F′.

We examined the multipotency of satellite cells. The cells were treated with commercially available adipogenic, cardiomyogenic and osteogenic inducing media. However, we found that satellite cells did not differentiate into adipocytes, cardiomyocytes and osteocytes – instead both BRE-KO and BRE-WT satellite cells rapidly differentiated in to myofibers directly after the differentiation media were replaced (Fig. S4). We decided to use a Wnt agonist (chemical structure in Fig. 8G) instead to induce osetogenic differentiation *in vitro*. Our Alizarin Red staining revealed the presence of Alizarin Red^+^ aggregates after 3 day Wnt agonist treatment in BRE-WT satellite cell cultures (Fig. 8D′). These cultures progressively generated larger aggregates of boney calcium deposition at day 7 (Fig. 8E′) and day 9 (Fig. 8F′). Furthermore, they were bigger than the Alizarin Red^+^ aggregates produced by BRE-KO satellite cell cultures (Fig. 8B′ and C′, respectively). Statistical analysis revealed that the average area of Alizarin Red^+^ calcium deposits in BRE-WT cultures was significantly larger than BRE-KO cultures at day 9 (Fig. 8H).

### Absence of BRE impairs satellite cell migration and chemotactic response

**Fig. 9.**
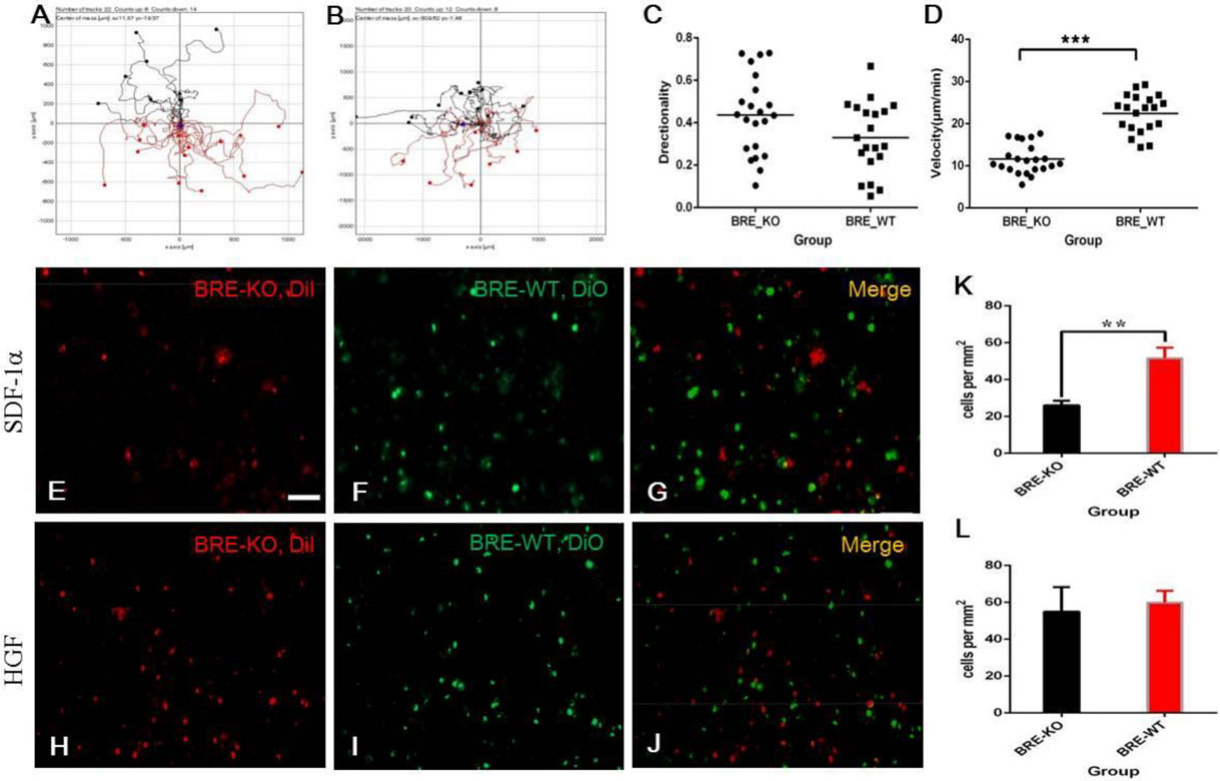
**(A-D) Time-lapse microscopy was used to record the migration of satellite cells *in vitro*.** Individual cell trajectory plots indicated that BRE-KO (A) and BRE-WT (B) migrated in all directions and there were no significant differences in directionality between the two groups (C). By contrast, there was a significant reduction in the velocity of BRE-KO satellite cells' movement compared with BRE-WT (D, ***P<0.001). (E-G) DiI-labelled BRE-KO (red, E) and DiO-labelled BRE-WT (green, F) satellite cells were tested for their abilities to chemotactic response to SDF-1α. (K) Statistical analysis revealed that BRE-KO satellite cells were significantly less responsive to SDF-1α than BRE-WT cells (**P<0.001). By contrast, when HGF was used as the chemoattractant (H-J), there was no difference in chemotactic response and migration (L). Data are presented as mean±s.e.m. Scale bars=100 μm.

Satellite cells migration is crucial for the recruitment of these cells to the injury site to facilitate muscle regeneration. We have used time-lapse microscopy to record the migration of BRE-KO and BRE-WT satellite cells *in vitro*. Images of the cells were captured at one minute intervals for a total duration of two hours. For both groups, 20 satellite cells were tracked and analyzed. The results indicated that both BRE-KO and BRE-WT satellite cells migrated in all directions as demonstrated by the individual cell trajectory plots (Fig. 9A and B, respectively), which were automatically calculated using an Image J software. There were no differences in directionality between BRE-KO and BRE-WT satellite cells (Fig. 9C). However, we found that in the absence of BRE, there was a significant reduction in the velocity of satellite cell migration (Fig. 9D).

It is has been reported that HGF and SDF-1α could chemotactically regulate satellite cell migration to the injury site (Suzuki et al., 2000; Ratajczak et al., 2003; Sherwood et al., 2004). We used a Multi-well Chemotaxis Chamber setup to examine the chemotactic response of BRE-KO and BRE-WT satellite cells to SDF-1α and HGF. The extent of cell migration was determined by the number of DiI-labelled BRE-KO (Fig. 9E and H for SDF-1α and HGF, respectively) and DiO-labelled BRE-WT (Fig. 9F and I for SDF-1α and HGF, respectively) satellite cells that have migrated across the membrane filter towards the chemoattractant. We established that there was no significant difference in BRE-KO and BRE-WT satellite cells' migratory response to HGF (Fig. 9L). By contrast, BRE-KO satellite cells were significantly less responsive to SDF-1α than BRE-WT cells (Fig. 9K).

### BRE protects CXCR4 from SDF-1α induced degradation

**Fig. 10.**
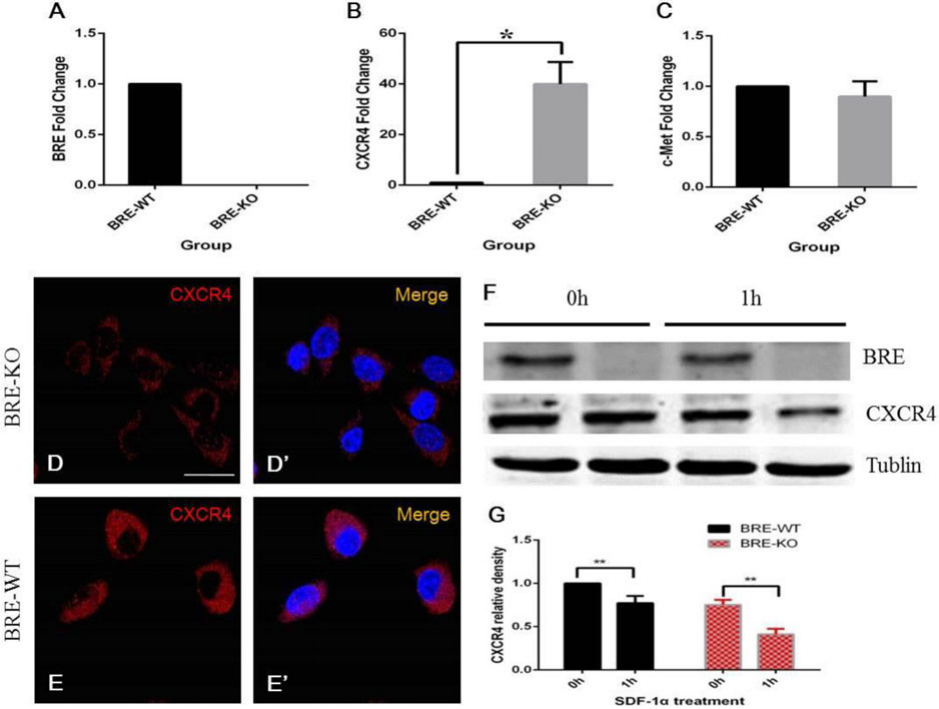
RT-qPCR analysis revealed that BRE-KO satellite cells expressed a significantly higher level of CXCR4 mRNAs (40 folds higher) than BRE-WT satellite cells (B, *P<0.05). (C) There was no significant difference in HGF receptors (c-Met) expression. (D–E′) Surprisingly, immunofluorescent staining indicated that BRE-WT satellite cells (E) were more intensely stained for CXCR4 expression than BRE-KO satellite cells (D). (F) Western blot analyses of CXCR4 were performed before and after 1 h SDF-1α treatment. (G) Statistical analysis revealed that CXCR4 was significantly degraded in both BRE-KO and BRE-WT satellite cells after 1 h SDF-1α treatment (**P<0.01). However, CXCR4 degradation in BRE-KO cells was significantly more rapid than in BRE-WT cell (50% vs 20%, respectively). Data are presented as mean±s.e.m. Scale bars=20 μm.

Above we have determined that BRE is required for cell migration and chemotactic response to SDF-1α but not HGF. CXCR4 is the receptor of SDF-1α, so we investigated CXCR4 expression in BRE-KO and BRE-WT satellite cells using RT-qPCR and immunofluorescent staining. Our RT-qPCR results revealed that BRE-KO satellite cells expressed a significantly higher level of *CXCR4* mRNAs (40 folds higher) than BRE-WT satellite cells (Fig. 10B). Surprisingly, at the protein level, immunofluorescent staining indicated that BRE-WT satellite cells (Fig. 10E) have higher protein level of CXCR4 expression than BRE-KO (Fig. 10D) satellite cells. These results appeared to contradict the RT-qPCR result because *CXCR4* mRNA was highly expressed on BRE-KO satellite cells but why were there less CXCR4 proteins in these mutant cells? We hypothesize that in the absence of BRE, CXCR4 protein was unstable and could easily be degraded. This would account for why more *CXCR4* mRNA and protein were synthesized to compensate for the CXCR4 protein degradation.

Previous studies have reported that after SDF-1α binds to CXCR4, the receptor is rapidly degraded through endocytosis and lysosomes sorting. To address our hypothesis above, we pre-treated satellite cells with cycloheximide (a protein biosynthesis inhibitor) for 30 min and then incubated the cells with growth culture medium containing 100 μg/ml SDF-1α. Total protein of these cells was isolated 1 h after treatment and CXCR4 expression was analyzed by western blot (Fig. 10F). The results showed that CXCR4 was degraded in both BRE-KO and BRE-WT satellite cells after 1 h SDF-1α treatment. However, CXCR4 degradation in BRE-KO cells was more rapid than in BRE-WT cells (Fig. 10G, 50% vs 20%, respectively). The results suggest that BRE normally protects CXCR4 from ligand-induced CXCR4 protein degradation.

## DISCUSSION

*Bre* was initially identified to be expressed in the brain, testis and ovary so that was how the name was derived. Presently, we have demonstrated that *Bre* is also expressed in the skeletal muscles and their associated satellite stem cells. It has been reported that *Bre* encodes a multifunctional protein that play a role in DNA repair (Dong et al., 2003), preventing apoptosis (Chan et al., 2008) and maintaining stemness (Chen et al., 2013). In this context, we were interested in *Bre*'s role in satellite stem cell physiology during skeletal muscle regeneration. It is now well establish that following skeletal muscle injury, the muscles repair itself by forming new myofibers derived from activated satellite cells (Musarò, 2014). When the muscles are injured, the satellite cells re-enter into the cell cycle and increase the number of available satellite cells for muscle regeneration. This event is associated with significant up-regulation of pax7 (marker for satellite cells) expression (Musarò, 2014). We analyzed BRE expression of in skeletal muscles after injury and found that, just like pax7, BRE expression was also significantly up-regulated. Hence, we hypothesized that *Bre* might play a key role in the control of skeletal muscle regeneration.

In this study, we use *Bre* knockout mice as a model to elucidate the role of *Bre* in skeletal muscle repair. We first confirmed that *Bre* was not expressed in our mutant mice at mRNA and protein levels. We were surprised to not find any phenotype in the *Bre* mutant mice despite reports that *Bre* was an important multifunctional gene. Moreover, the BRE-KO skeletal muscles appeared histological similar to BRE-WT mice. We asked whether the effect of the mutation was more obvious at the cellular and molecular level following experimental intervention. Therefore, we chemically damaged the skeletal muscles in BRE-KO mice to determine whether muscle regeneration was affected as compared with BRE-WT mice. We found that there were significantly fewer BRE-KO satellite cells present at the injury site and also the new formed myofibers were smaller during the early stages of muscle repair, as compared with their normal counterpart. It is well established that normally after muscle injury, satellite cells proliferate and migrate into the damage site where they fuse together to form new myofibers. These myofibers then become interconnected with intrinsic intact myofibers present around the injury site (von Maltzahn et al., 2013). This suggests that *Bre* might be required for satellite cell proliferation, migration and differentiation. Hence, we investigated these key biological processes using satellite cells *in vitro*.

*In vitro*, we determined that without BRE, satellite cell fusion was deficient. Specifically, we demonstrated a decreased in satellite cell fusion index and shorter myofibers formed as compared with BRE-WT satellite cells. The relation between BRE and satellite cell fusion is unclear but satellite cell differentiation involves this process and an increase in MyoG expression. We found MyoG expression was significantly repressed in BRE-KO satellite cells. MyoG is a member of the myogenic regulatory transcription factors and an important regulator for skeletal muscle development and regeneration. MyoG has a highly restricted expression pattern and found in primary myotome of mouse embryo at E9 (Sassoon et al., 1989), newly regenerated myofibers (Zhao et al., 2002) and differentiated satellite cells. The MyoG expression is thought to be regulated by Notch, Wnt and FGF signaling pathways (Chapman et al., 2006; Borello et al., 2006; Buckingham, 2006), protein kinase A (Li et al., 1992a) and protein kinase C (Li et al., 1992b). It is also believed that a correct spatio-temporal switch from Notch to Wnt signaling was crucial for efficient skeletal muscle regeneration (Brack et al., 2008). We believe that BRE is required for proper MyoG expression during skeletal muscle regeneration. In the absence of BRE, satellite cell fusion and differentiation could occur but retarded, probably caused by an inadequate increase in MyoG expression.

Satellite cells are regarded as being the stem cells of skeletal muscles, with the ability of undergoing self-renewal. They are also thought to be multipotent and can be induced to differentiate into multiple cell lineages, including adipocytes and osteocytes, using adipogenic inducing medium and bone morphogenetic proteins, respectively (Asakura et al., 2001; Ozeki et al., 2006). Satellite cells normally differentiate into myofibers very rapidly when cultured in myogenic differentiation medium but can also transdifferentiate into osteocytes when induced (Kocić et al., 2012). Amongst the known signaling pathways that regulate osteoblast development, Wnt signaling is thought to be the most crucial. Normally, Wnt binds to the Frizzled receptor to phosphorylate the GSK3/Axin complex that stabilizes intracellular β-catenin. The β-catenin translocates into the nucleus and could activate genes associated with osteogenic differentiation, such as Runx2. In this study, we have used a small molecule Wnt agonist that mimics the effects of Wnt but without inhibiting GSK-3β (Liu et al., 2005), to induce the osteogenic differentiation in satellite cells. We established that osteogenic induction was very rapid and Alizarin red stained osteoblasts were discernable in the cultures after 3 days whereas classical osteogenic inducing medium requires approximately 2 weeks. In addition, we determined that osteogenic differentiation could also occur in BRE-KO satellite cells but significantly deficient as compared with their normal counterpart. Previously, we have reported that BRE acts like an adaptor protein that promotes stemness and at the same time inhibits the differentiation in human umbilical cord perivascular (HUCPV) mesenchymal stem cells (Chen et al., 2013). Moreover, we silenced BRE expression and revealed that HUCPV cells accelerated their osteogenic differentiation after induction. This would correlate with our present observations that osteogenic differentiation is deficient in BRE-KO satellite cells. However, BRE does not appear to be required for maintaining stemness in satellite cells, as was the case for HUCPV cells. This is because *in vitro*, we demonstrated that BRE-KO and BRE-WT satellite cells could proliferate (self-renewal) at similar rates and can both transdifferentiate into osteocytes (but with different efficiency). Nevertheless, it does affect differentiation and may explain why myogenic cell fusion and differentiation is impaired when skeletal muscles are injured in BRE-KO mice.

Chemokines, like SDF-1α, are important for regulating cell migration. Normally SDF-1α is mainly expressed in the bone marrow (Bleul et al., 1996) but is also produced by the skeletal muscles when they are injured. SDF-1α acts on cells that express the CXCR4 receptor and is involved in regulating the migration of transplanted cells in damaged muscles (Grabowska et al., 2012). We compared the ability of BRE-KO and BRE-WT satellite cells to express CXCR4 and determined that BRE-KO cells expressed a significantly higher level of *CXCR4* transcripts but a slightly lower level of the CXCR4 protein. It has been reported that the correlation between mRNA and protein expression can be as little as 40%. This poor correlation could be attributed to post-transcriptional and translational regulations, protein stability and protein modification (de Sousa Abreu et al., 2009). In this context, we believe that in the absence of BRE, the CXCR4 receptor is more readily degraded. Ubiquitination is the process where ubiquitin become attached to target proteins destined for destruction – which is crucial in many biological processes. It has been reported that cell surface CXCR4 receptor is a target for ubiquitin (Saini et al., 2010) and ubiquitination of CXCR4 leads to endocytosis, endosome sorting and subsequent degradation (Marchese and Benovic, 2001). Nevertheless, the exact mechanisms involved are still not fully understood (Holleman and Marchese, 2014). It has been demonstrated that three enzymes are involved which includes ubiquitin-activating enzymes (E1s), ubiquitin-conjugating enzymes (E2s), and ubiquitin ligases (E3s). These are needed for ubiquitin attachment to the target substrate proteins. AIP4 (a Nedd4-like E3 ubiquitin ligase) together with Hrs and Vps4, coordinate the ubiquitination and deubiquitination pathways that mediate in the degradation of CXCR4 (Marchese et al., 2003). BRE is a component of the BRCA1-A complex which is an ubiquitin E3 ligase complex possessing deubiquitinase activity that specifically removes Lys-63-linked ubiquitin on histones H_2_A and H_2_AX. Hence, we hypothesize that BRE could mediate in CXCR4 turnover by regulating their deubiquitination. In the absence of BRE, CXCR4 is more rapidly degraded which explains why BRE-KO satellite cells produced significantly higher levels of CXCR4 mRNAs (to compensate) than BRE-WT satellite cells. In addition, the lack of CXCR4 clarifies why BRE-KO satellite cells were significantly less responsive to chemoattractant SDF-1α than BRE-WT satellite cells in our chemotactic migration assays.

**Fig. 11.**
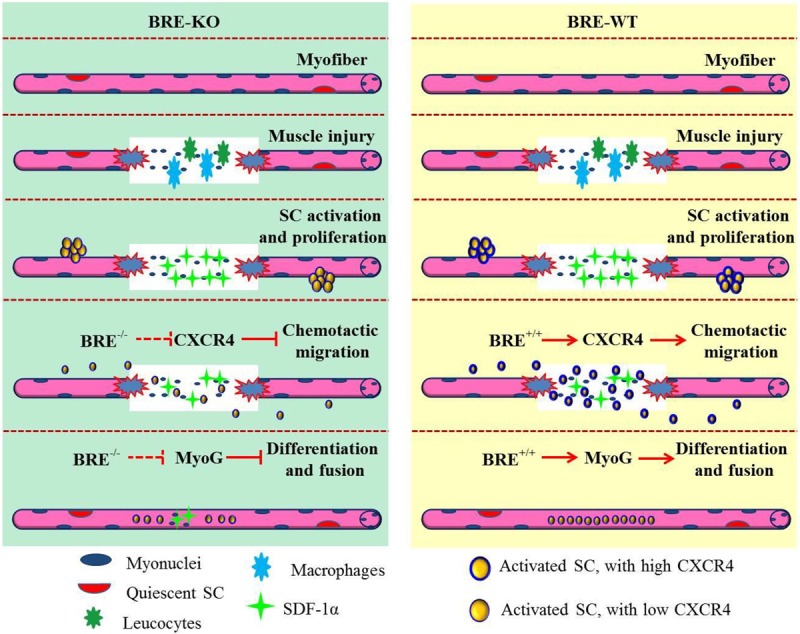
**Proposed mechanism for *Bre* function in the regulation of skeletal muscle regeneration.** We propose that *Bre* null mutation impairs skeletal muscle regeneration by reducing CXCR4 expression in satellite cells. This subsequently represses satellite cell migration to the muscle injury site. The *Bre* mutation also represses MyoG expression during satellite cell differentiation and fusion which further negatively impact on skeletal muscle repair.

In conclusion, our data suggests that BRE is required for efficient satellite stem cell migration, homing and differentiation during skeletal muscle regeneration. In addition, BRE is required for maintaining CXCR4 expression and may explain why satellite stem cell migration is deficient in the mutant satellite stem cells. We have proposed a model illustrating BRE's role in skeletal muscle regeneration in Fig. 11.

## MATERIALS AND METHODS

### Production of *Bre* knockout mice

Homozygous BRE-KO mice were generated using the Cre/loxP system on a C57/BL/6J background. Male heterozygous PGCs (primordial germ cells)-Cre transgenic mice (129-Alpltm1 (cre) Nagy/J, Stock Number: 008569), where the Cre recombinase sequence was knock-in into the *TNAP* (tissue non-specific alkaline phosphatase) gene. These mice (from here on referred to as *TNAP^Cre/+^*) were purchased from the Jackson Laboratory. Female *Bre*-targeted mice (B6Dnk; B6N-Bretm1a (EUCOMM) Wtsi/H), where the exon3 are flanked by two loxP sites, were acquired from the International Knockout Mouse Consortium. These mice from here on are referred to as *BRE^fx/fx^*. BRE-KO mice were generated by crossing male *TNAP^Cre/+^* mice with female *BRE^fx/fx^* mice. All animal handling and surgical procedures have been approved by the Animal Research Ethics Committee of Chinese University of Hong Kong and Hong Kong department of health.

### Cardiotoxin-induced muscle injury model

BRE-WT and BRE-KO mice were anesthetized by intraperitoneal injection of katamine (75 mg/kg) and xylazine (7.5 mg/kg). We selected the tibialis anterior (TA) muscle in the leg for experimentation. The TA in one of the legs was injected with 10 μl of 100 μM cardiotoxin (CTX; Sigma-Aldrich) while the contralateral control leg was injected with saline. The TA muscles were harvested for analysis on day 4, 7 and 15 after cardiotoxin-induced injuries. The muscles harvested were used for mRNA and protein isolation, routine histology and immunohistochemistry staining.

### Satellite cells culture

Satellite cells were isolated from both BRE-WT and BRE-KO mice using a modified protocol developed by Sherwood et al. (2004). Briefly, the *extensor digitorum longus* (EDL) muscle was dissected intact with the tendon attached from both BRE-WT and BRE-KO mice. The EDL muscles were then digested in 0.2% collagenase type I for 25-30 min at 37°C. Following collagenase digestion, individual myofibers were released from the muscle mass by repeatedly trituration in warm culture medium. The single myofibers were then transferred into culture dishes coated with matrigel. These myofibers were cultured in DMEM-F12 medium supplemented with 20% fetal bovine serum, 10%, horse serum, 1% chicken embryo extract, 10 ng/ml bFGF and 1% Penicillin/Streptomycin. After 1 week of culture, the satellite cells have migrated away from the myofibers and onto the culture dish. We performed serial pre-plating of the cells that have invaded these dishes. These cells were trypsinized into a cell suspension and then serially pre-plated onto uncoated culture dishes for 2 h at 37°C in 5% CO_2_ to remove the more adhesive fibroblasts. The purity of the isolated satellite cells was assessed by immunocytochemistry for the satellite cell specific marker, pax7. In addition, these satellite cells were genotyped to confirm that they do not express *Bre*.

### Immunofluorescent staining

Cells maintained on glass cover-slips were fixed and permeabilized in 0.5% Triton X-100 in PBS for 1 h at room temperature. These cells were then blocked with 2% BSA and 5% host serum in PBS for 1 h. For skeletal muscles, the section were dewaxed, hydrated and microwaved for 15 min in 10 mM citric acid-sodium citrate buffer (pH 6.0) for antigen retrieval. Following five washings in PBS, the sections were blocked with 5% normal goat serum in PBS for 30 min at 37°C. These specimens were incubated with the desired primary antibodies diluted in 1% BSA and 2.5% host serum in PBS overnight at 4°C. The specimens were then washed five times in 0.05% Tween-20/PBS and incubated with the appropriate Alexa Fluor conjugated secondary antibody diluted in 1% BSA for 1 h at 4°C. The cells were washed in PBST, counterstained with 4 μg/ml DAPI, mounted in 70% glycerol and examined under a confocal microscope (Olympus FV1000 ZCD). The samples were photographed and analyzed using a FV10-ASW 1.7 viewer software. The primary antibodies used were: Rabbit anti-BRE (Cell Signaling, 1:200); Mouse anti-Pax7 (Developmental Studies Hybridoma Bank, 1:100); Rabbit anti-CXCR4 (Santa Cruz, 1:50) and Mouse anti-BrdU (ZYMED, 1:100). The secondary antibodies used were: Alexa Fluor 488 donkey anti-mouse IgG (Invitrogen, 1:300) and Alexa Fluor 647 donkey anti-rabbit IgG (Invitrogen, 1:300).

### Transmission electron microscopy

Satellite cells were pelleted and then harvested in 0.1 M sodium cacodylate buffer (pH 7.4). The cell pellets were then fixed in 0.25% glutaraldehyde plus 2% paraformaldehyde for 2 h at room temperature and washed 3 times in sodium cacodylate buffer. The cells were post-fixation in 1% OsO_4_ for 1 h and washed with distilled water. The samples were then dehydrated in ethanol and embedded in Epson resin (Epon-812, DDSA, MNA and DMP-30). The embedded cell pellets were sectioned at 70 nm using a diamond knife and the sections were post stained with 4% uranyl acetate and lead citrate buffer. All stained sections were viewed under a Hitachi H7700 Transmission Electron Microscope.

### Scanning electron microscopy

Satellite cells were cultured on glass coverslips to 80% confluence and then fixed in 2.5% glutaradehyde. The cultures were washed three times in PBS and post-fixed in 1% osmium tetraoxide made up in distilled water. The cultures were then dehydrated in ethanol, critical dried and gold-coated. All specimens were viewed under a Hitachi SU8010 Scanning Electron Microscope with i-XRF EDS system.

### Cell proliferation assays

BRE-WT and BRE-KO satellite cells were cultured on matrigel coated culture dishes at low cell density and pulsed labelled with growth medium supplemented with 10 μM BrdU (Amersham Biosciences) for 3 h. The cultures were then washed three times in fresh growth medium and fixed in 4% paraformaldehyde for 30 min. To visualize the BrdU-labelling, the cultures were permeabilized with 2 M HCl+0.5% Triton X-100 in PBS for 30 min at room temperature. The samples were double immunofluorescently stained with BRE and BrdU antibodies along with the appropriate fluorescent-labelled secondary antibodies. The stained samples were analyzed with an Olympus FV1000 ZCD laser scanning confocal system. The percentage of proliferating (BrdU^+^) satellite cells were determined and statistically compared between the BRE-WT and BRE-KO groups.

### Myogenic differentiation assay

Satellite cells from BRE-WT and BRE-KO EDL muscles were cultured on matrigel coated culture dishes to 80% confluence. The medium was then replaced with myogenic differentiation medium containing DMEM F12 supplemented with 10% Horse serum and 1% PS. The cells were incubated for 24-36 h, fixed in 4% paraformaldehyde and stained with DAPI. All samples were photographed under a Nikon Ti-E microscopy. The percentage of nuclei within myofibers and the mean length of myofibers were measured and analyzed using an Image J software.

### Osetogenic differentiation assay

We tested the multipotency of BRE-WT and BRE-KO satellite cells. The satellite cells were cultured on matrigel coated dishes to 90% confluence. Then commercial available osteogenic, adipogenic or cardiomyogenic differentiation medium were added to the cultures according to the manufacturer's instructions (Cat.No.A10072-01, A10070-01, A25042SA, Life Technologies). We determined that these differentiation media were unable to induce satellite cells to differentiate into adipocytes, osteocytes and cardiomyocytes. Consequently, we used growth medium supplement with 10 μM Wnt agonist (CalBiochem, Cat no: 681665) to induce osteogenesis. The medium was changed every 2 days and after 3-9 days incubation, the cells were fixed with 4% paraformaldehyde and stained with Alizarin Red S dye. This dye reveals the presence of calcium phosphate deposits normal found in bone matrix. The stained cultures were photographed under an Olympus IX83 Inverted Microscope with ZDC. Regions in the cultures containing Alizarin Red^+^ calcium deposits were measured quantified and statistically analyzed using SPSS.

### Time-lapse migration analyses

BRE-WT or BRE-KO satellite cells were allowed to attach to confocal dishes in culture medium containing HEPES buffer. The dishes were sealed with para-film to prevent evaporation and the dishes were housed in a heated humidified chamber at 37°C. The movement of individual cells was recorded and tracked using a Nikon Ti-E live cell imaging system. For time-lapse recording, an image was automatically captured at 1 min intervals for a total duration of 2 h. The data were saved as multipage TIFF files and analyzed using Image J software. Briefly, the TIFF files were loaded into the Image J software with ‘Manual Tracking’ plug-in for tracking the movement of individual cells. This was then followed by data analysis using the ‘Chemotaxis and Migration tool’ plug-in to calculate the velocity and directionality of individual cell movement. For each experimental group, the movement of up to 20 satellite cells was analyzed.

### Chemotactic migration assay

Chemotactic migration assays were performed using a Neuro Probe Reusable Multi-well Chemotaxis Chambers, according to manufacturer's instructions. BRE-WT and BRE-KO satellite cells were labelled with fluorescent dyes Vybrant DiO and DiI, respectively. The fluorescently labelled satellite cells were detached from the culture dishes with trypsin and suspended in culture medium. Both BRE-WT and BRE-KO satellite cells (at exactly the same cell concentration) were mixed together to give a final concentration of 1×10^6^ cells/ml for each cell type. The polycarbonate membrane used in the Multi-well Chemotaxis Chambers was pre-coated with matrigel and contained numerous 10 μm pores. 50 μl of the cell suspension was loaded onto the upper wells of the chamber while 25 μl of culture medium containing 1 μg/ml of SDF-1α or 1 μg/ml of HGF was introduced to the lower wells. After incubation at 37°C for 12 h, cells that have not migrated and remained on top of the membrane were carefully wiped off using sterile cotton buds. The lower surface of membrane directly opposing the chemoattractants was examined under a fluorescent microscopy and photographed. The number of DiO-labelled BRE-WT and DiI-labelled BRE-KO satellite cells were counted, recorded and analyzed for each well.

### Ligand-induced receptor degradation assay

BRE-WT and BRE-KO satellite cells were grown on culture dishes coated with matrigel coated to approximately 80% confluence. The cells were cultured in medium containing 20 μg/ml of cycloheximide for 30 min at 37°C and 5% CO_2_. The cells were then washed with fresh culture medium three times (10 min each) and SDF-1α was added with a final concentration of 100 μg/ml. After 1 h incubation, the satellite cells were washed with culture medium three times, washed in PBS and pelleted for protein extraction and western blot analysis.

### Western blot analysis

Total protein was extracted from muscle tissues and cultured satellite stem cells with RIPA buffer supplemented with protease inhibitor cocktail and 1 mM PMSF. For muscle tissues, after adding lysis buffer, the samples were dissected into small pieces and homogenized with an electric homogenizer. For satellite cells, it was pelleted, collected and then added with lysis buffer. The protein concentration was measured with a BSATM Protein Assay Kit (Pierce). The protein extract was resolved in a 10% SDS-PAGE gel, transfer onto a nitrocellulose membrane and blocked with 5% nonfat dry milk in TBST (0.1% Tween-20 in TBS) for 2 h at room temperature. Primary antibody diluted with blocking buffer was added to the membrane and incubated overnight at 4°C. The treated membrane was then washed five times in TBST (5 min each) and treated with the appropriate secondary antibody for 1 h at room temperature. After washing with TBST, the membrane was examined using an Odyssey Infrared Imaging System.

The antibodies used were: Rabbit anti-BRE (Cell Signaling, 1:1000); Mouse anti-Pax7 (Developmental Studies Hybridoma Bank, 1:1000); Rabbit anti-CXCR4 (Santa Cruz, 1:200); Mouse anti-GAPDH (Sigma, 1:15,000); Rabbit anti-MyoD (Santa Cruz, 1:200); Mouse anti-Tublin (Santa Cruz, 1:15,000); Donkey anti-mouse IDRye 800CW (Licor Bioscience, 1:15,000); Donkey anti-rabbit IDRye 680CW (Licor Bioscience, 1:15,000).

### RT-qPCR analysis

Total RNA was extracted from muscle tissue or satellite cells using an RNeasy Fibrous Tissue Mini Kit or RNeasy Mini Kit (Qiagen) respectively, according to the manufacturer's protocol. cDNA was synthesized using RevertAid First Strand cDNA Synthesis Kit (Thermo Scientific) with Oligo dT 18 primer. Quantitative Real-time PCR was performed with SYBR Premix Ex Taq Kit (TAKARA). The quantification of mRNA expression was performed with an ABI ViiA 7 Real Time PCR System. The primers used in the study are listed in Table S1.
